# Development of a Margin Determination Framework for Tumor-Tracking Radiation Therapy With Intraoperatively Implanted Fiducial Markers

**DOI:** 10.3389/fonc.2021.753246

**Published:** 2021-10-07

**Authors:** Jihun Kim, Min Cheol Han, Jee Suk Chang, Chae-Seon Hong, Kyung Hwan Kim, Hwa Kyung Byun, Ryeong Hwang Park, Woong Sub Koom, Seong Yong Park, Jin Sung Kim

**Affiliations:** ^1^ Department of Radiation Oncology, Yonsei University College of Medicine, Seoul, South Korea; ^2^ Department of Radiation Oncology, Yonsei Cancer Center, Seoul, South Korea; ^3^ Department of Thoracic and Cardiovascular Surgery, Yonsei University College of Medicine, Seoul, South Korea

**Keywords:** lung metastasis, intraoperative fiducial marker implantation, CyberKnife Synchrony, fiducial tracking, internal target volume, stereotactic ablative radiotherapy

## Abstract

**Purpose:**

To develop an internal target volume (ITV) margin determination framework (or decision-supporting framework) for treating multiple lung metastases using CyberKnife Synchrony with intraoperatively implanted fiducial markers (IIFMs). The feasibility of using non-ideally implanted fiducial markers (a limited number and/or far from a target) for tracking-based lung stereotactic ablative radiotherapy (SABR) was investigated.

**Methods:**

In the developed margin determination framework, an optimal set of IIFMs was determined to minimize a tracking uncertainty-specific ITV (ITV_tracking_) margin (margin required to cover target-to-marker motion discrepancy), i.e., minimize the motion discrepancies between gross tumor volume (GTV) and the selected set of fiducial markers (FMs). The developed margin determination framework was evaluated in 17 patients with lung metastases. To automatically calculate the respiratory motions of the FMs, a template matching-based FM tracking algorithm was developed, and GTV motion was manually measured. Furthermore, during-treatment motions of the selected FMs were analyzed using log files and compared with those calculated using 4D CTs.

**Results:**

For 41 of 42 lesions in 17 patients (97.6%), an optimal set of the IIFMs was successfully determined, requiring an ITV_tracking_ margin less than 5 mm. The template matching-based FM tracking algorithm calculated the FM motions with a sub-millimeter accuracy compared with the manual measurements. The patient respiratory motions during treatment were, on average, significantly smaller than those measured at simulation for the patient cohort considered.

**Conclusion:**

Use of the developed margin determination framework employing CyberKnife Synchrony with a limited number of IIFMs is feasible for lung SABR.

## Introduction

CyberKnife Synchrony, a fiducial-based tracking method, is an attractive radiation treatment (RT) modality with CyberKnife’s dosimetric advantages due to non-coplanar delivery of radiation doses (1). In general, CyberKnife Synchrony requires fiducial marker (FM) implantation prior to RT simulation and planning, which is mainly performed *via* a percutaneous, computed tomography (CT)-guided insertion procedure. Moreover, FMs can be inserted during surgery (2). Intraoperative FM implantation can be beneficial in some clinical situations, in which the extent of surgery is limited by concerns on morbidity or pulmonary function. In this case, the remaining unresectable lesions can be treated *via* stereotactic ablative radiotherapy (SABR) with intraoperatively implanted FMs (IIFMs). Furthermore, when a “missing lesion” exists (3), which is not detectable during surgery, FMs can be intraoperatively implanted and used for the following SABR. In addition to the use of IIFMs, existing FMs that were used in previous treatments can be further utilized for additional RT.

These challenging clinical scenarios, in which a limited number of FMs are available, including those located far from a target, pose the following clinical questions because inserting three to five FMs near each radiation target is ideal for the CyberKnife fiducial tracking-based RT: 1) which among the various RT techniques, including CyberKnife fiducial tracking, respiratory gating, and internal target volume (ITV)-based technique, is the optimal treatment?, 2) if a lesion is treated using CyberKnife fiducial tracking, how can the uncertainty related to motion discrepancy between the target and existing FMs be quantified for consideration in treatment planning?

Therefore, to address the aforementioned and unanswered questions, we aimed to develop a margin determination framework (a decision-supporting framework) and evaluate its clinical feasibility. Specifically, this study evaluated the feasibility of using the developed margin determination framework for treating multiple lung metastases using IIFMs. To the best of the authors’ knowledge, this study is the first to make an effort towards developing such a novel framework, which has the potential to facilitate usage of non-ideally implanted FMs for tracking-based RT, thereby reducing the risk of side effects by additional FM implantation. Furthermore, successful implementation of the developed framework can facilitate multi-site SABR using a limited number of FMs as dynamic transition into repeat oligometastatic and induced oligometastatic diseases may be possible depending on the response to local or systemic therapy (4).

## Methods and Materials

### ITV_tracking_ Margin Determination Framework

The developed margin determination framework was designed to 1) analyze the motions of a radiation target and IIFMs, 2) evaluate the motion synchronization level between the target and all possible sets of the IIFMs, and 3) suggest an optimal FM set *S* ^*^ showing maximum synchronization with an additional margin required to cover the target-to-marker motion discrepancy. This additional margin is referred to as “tracking uncertainty-related ITV (ITV_tracking_) margin” in this study. The margin determination framework was developed using MATLAB (MathWorks, Natick, MA, USA). The steps in the developed framework can be mathematically formulated as follows:


(1) $$S^{*}=\arg\min f(S),\;S\subset \left\{1,2,\text{...},N_{F}\right\}$$

(2) $$f(S)={\left||{\text{u}}_{T}-{\text{u}}_{S}|\right|}_{2}$$

where *f(S)* represents the root mean square difference between the target motion **u** *_T_* and the average motion **u** *_S_* of a subset of the FMs *S*, and *N_F_* represents the number of the IIFMs. Finally, ITV_tracking_ margin is defined as follows:


(3) $$\text{IT}{\text{V}}_{\text{tracking}}\text{ margin}=\left||{\text{u}}_{T}-{\text{u}}_{S^{*}}|\right|$$

### Fiducial Marker Tracking Algorithm

A template matching-based registration algorithm was developed to calculate the motions of each of the IIFMs in 4D CT using ITK (5). FMs were initially detected by thresholding HU values (HU > 1500). Then, a template matching algorithm was used to find an optimal translation vector that maximizes a mutual information (6–8) between the reference (0% phase) and target (10–90% phases) images. A template was defined as a cubic region of 4 × 4 × 10 mm^3^ centered at the detected FM on the reference image; the dimension of the cubic region-of-interest was optimally determined by a parametric study. Multi-resolution template matching was performed by starting with a grid size of 1.0 mm and reducing the grid spacing to 0.25 mm.

### Phantom Study

A phantom study was conducted to evaluate the feasibility of using 4D CT and the developed registration-based FM tracking method for the calculation of FM motion. The FM motions on 4D CT images were analyzed using two methods, manual and automatic (template matching-based), and were compared with the known motions. A Quasar Respiratory Motion Phantom (Modus QA, London, Ontario, Canada) was used to simulate a respiratory motion (9), expressed by *z*(*t*) = *z*_0_ – *b* cos^4^(*πt*/*τ*), where *z*(*t*) and *z*_0_ represent the inferior-superior (I-S) location of the target at time *t* and initial position, respectively, *b* represents the peak-to-peak motion amplitude, and *τ* represents the respiration cycle (*b* = 5, 10, 15, and 20 mm; *τ* = 4 s). Three gold FMs (approximately 1 mm diameter and 3 mm length) were inserted into a Cedar Lung Tomour Insert (Modus QA, London, Ontario, Canada). Ten-phase 4D CTs of the phantom were acquired using a Toshiba Aquilion CT simulator (Toshiba Medical System, Japan) and Abches (APEX Medical, Inc., Japan), a respiration-monitoring device, with a slice thickness of 3 mm. It is noted that this 4D CT acquisition protocol used for the phantom was the same as that in the patient 4D CT acquisition.

### Patient Study

**Figure 1:**
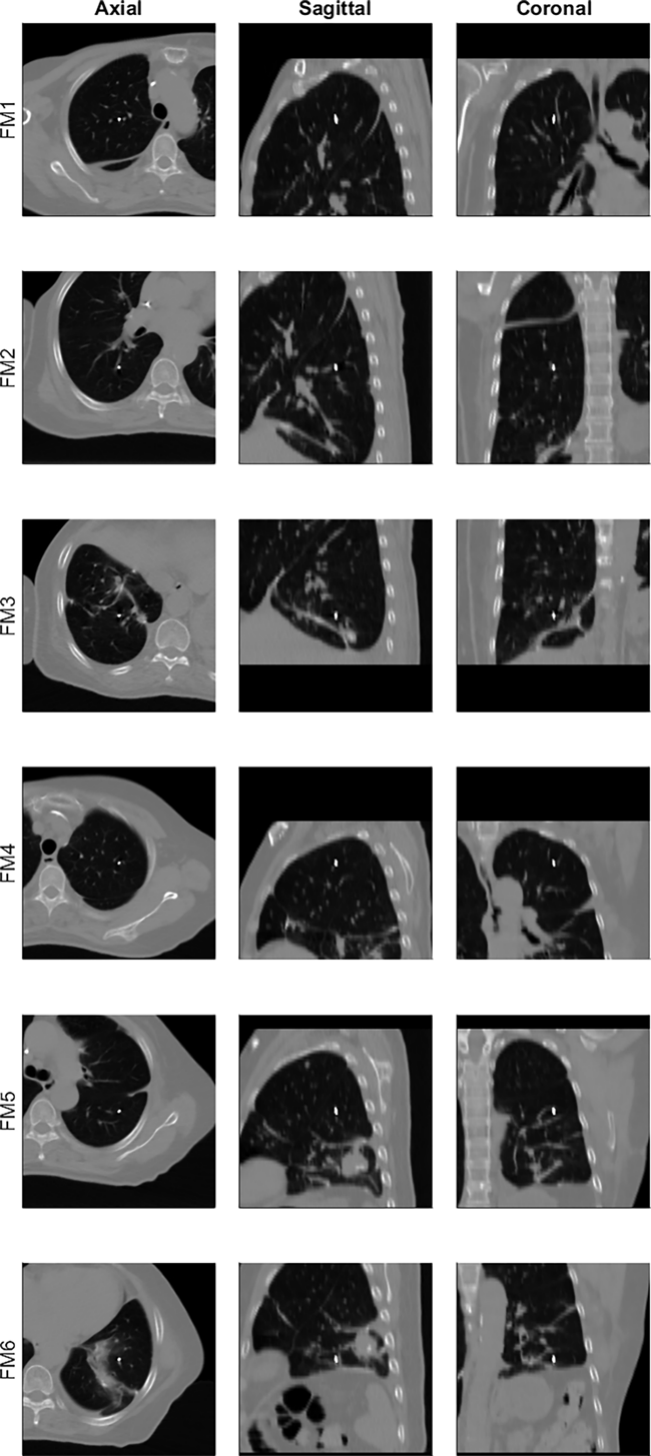
Example of intraoperatively implanted fiducial markers (six markers for patient 14), each of which is displayed at the center of the axial (first column from left), sagittal (second), and coronal (third) views.

Seventeen patients with lung metastases and IIFMs were considered for a retrospective analysis approved by the institutional review board of Severance Hospital, Seoul, South Korea (4-2021-0440). Characteristics of the patients are summarized in **Table 1**. The number of lung lesions varied among the patients (range: 1 to 9). Two to six FMs (median: 5) were implanted for each patient during surgery performed prior to the RT of the lung metastases. Maximally, three FMs were implanted into each side of the lungs, resulting in a total of six FMs. **Figure 1** presents a patient case with six IIFMs. As illustrated in **Figure 1**, in general, one FM was implanted into each of the upper, middle, and lower lobes on each side the lungs (in total, 73 IIFMs for 17 patients). Lung lesions of the patient cohort were treated with one of the following dose-fractionation schemes: 20 Gy × 1, 25 Gy × 1, 30 Gy × 1, 18 Gy × 3, and 6 Gy × 5. Continuous positive airway pressure (CPAP) with a pressure of 12-17 cmH_2_O (median: 15 cmH_2_O) was used for respiration motion management as it has been shown to effectively reduce radiation dose to the lung (10–12).

**Table 1:** Summary of patient characteristics.

Patient #	Number of GTVs			Number of fiducial markers			Number of CK plans	Number of log-available CK plans
	Right	Left	Total	Right	Left	Total		
1	6	3	9	3	2	5	9	8
2	1	0	1	3	0	3	1	1
3	0	2	2	3	2	5	2	2
4	0	1	1	3	2	5	1	1
5	1	0	1	2	2	4	1	1
6	4	0	4	3	3	6	4	3
7	1	0	1	2	1	3	0	0
8	3	1	4	2	1	3	4	3
9	2	0	2	2	2	4	2	2
10	1	0	1	1	1	2	1	0
11	1	1	2	2	2	4	2	2
12	1	0	1	3	0	3	1	1
13	1	0	1	3	2	5	1	1
14	2	2	4	3	3	6	4	4
15	1	0	1	3	2	5	1	1
16	4	1	5	3	2	5	5	1
17	2	0	2	3	2	5	2	0
Total	31	11	42	44	29	73	41	31

GTV, gross tumor volume; CK, CyberKnife.

The developed margin determination framework was tested for the patient data. For each of the lung targets, the motion-to-marker motion discrepancies were analyzed, finally resulting in an optimal set of IIFMs with a corresponding ITV_tracking_ margin. The accuracy of the developed FM tracking algorithm was evaluated by comparing the automatically registered motions with the manually measured motions for all the 73 FMs.

The feasibility of the developed margin determination framework was further evaluated by comparing the motions of the IIFMs calculated using the 4D CT with the in-treatment motions calculated using treatment log files. Using the three-dimensional coordinates of the FMs tracked during the treatment beam delivery (average motion of the FMs), motion amplitudes during each treatment fraction were calculated. A paired two-tailed t-test with 95% confidence was conducted using MATLAB to evaluate the hypothesis that the pretreatment and average in-treatment motions of the FMs were statistically different.

## Results

### ITV_tracking_ Margin Calculation

**Figure 2:**
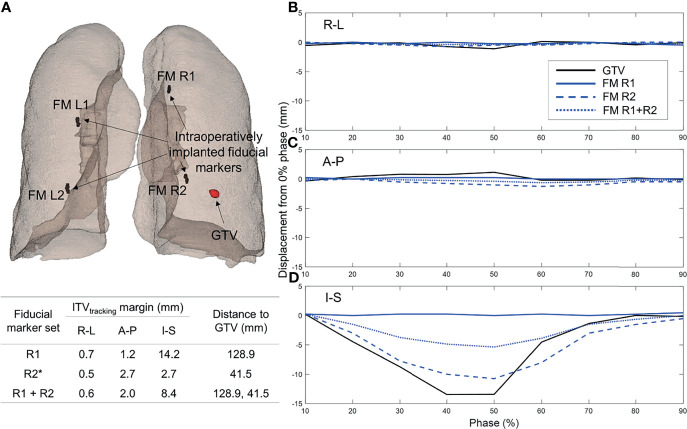
ITV_tracking_ determination results: **(A)** illustrative example of the processing to optimally select a fiducial marker (FM) set with a minimal ITV_tracking_ margin by comparing the motions of the GTV and all possible sets of the fiducial markers, **(B–D)** histograms of the ITV_tracking_ margins (i.e., the motion differences between the gross tumor volume and optimally selected FM set) calculated across 42 lung lesions from 17 patients in the **(B)** R-L, **(C)** A-P, and **(D)** I-S directions, respectively. ITV, internal target volume; R-L, right-left; A-P, anterior-posterior; I-S, inferior-superior; CK, CyberKnife.

**Figure 2A** illustrates how the developed framework determined an optimal FM set with a minimal ITV_tracking_ margin. Consequently, for patient 5, to treat the GTV with the FM R2, ITV_tracking_ margins of 0.5, 2.7, and 2.7 mm were required to cover the GTV-to-marker motion discrepancies in the right-left (R-L), anterior-posterior (A-P), and I-S directions, respectively.

The ITV_tracking_ margins calculated across all of the lung lesions were 1.0 ± 0.8, 1.1 ± 1.2, and 1.8 ± 1.7 mm in the R-L, A-P, and I-S directions, respectively (see **Figures 2B–D**). Of the 42 lung lesions, only one required an ITV_tracking_ margin larger than 5 mm (8.4 mm in the I-S direction for patient 7). This lesion was treated with a volumetric-modulated arc therapy (VMAT) plan with the ITV technique. Detailed information (selected FMs, corresponding ITV_tracking_ margins, and GTV-to-marker distances) of the ITV_tracking_ determination results for all lung lesions is summarized in **Table 2**. The GTV-to-marker distance was 59.3 ± 24.6 mm.

**Table 2:** Summary of the ITV_tracking_ determination results for each of the 42 lung lesions for 17 patients.

Patient #	GTV #		Optimal FM set	ITV_tracking_ margin (mm)			GTV-to-FM distance (mm)
				R-L	A-P	I-S	
1	*Right*	1	R3	0.7	0.2	1.0	30.9
		2	R2, R3	1.4	0.9	0.6	69.7, 20.3
		3	R2, R3	0.9	0.6	0.7	42.7, 33.9
		4	R1	1.2	2.8	3.8	103.7
		5	R2, R3	1.3	0.5	0.8	18.7, 68.5
		6	R1, R2	1.0	0.7	1.3	90.4, 38.8
	*Left*	1	L1, L2	3.2	0.9	0.3	52.7, 59.3
		2	L1, L2	0.7	0.8	2.0	100.8, 11.1
		3	L2	3.3	4.1	4.3	42.0
2	*Right*	1	R2	0.4	0.1	0.4	67.9
3	*Left*	1	L1	0.9	1.9	2.1	39.4
		2	L1	1.0	1.2	2.9	63.0
4	*Left*	1	L2	1.2	0.2	2.9	20.8
5	*Right*	1	R2	0.5	2.7	2.7	41.5
6	*Right*	1	R1	0.8	0.1	4.8	45.5
		2	R1, R2	1.5	0.8	0.4	128.5, 52.6
		3	R3	0.3	0.1	4.9	55.6
		4	R2	0.4	0.2	0.4	47.9
7	*Right*	1	R2	0.4	1.1	8.4	52.4
8	*Right*	1	R1, R2	1.3	1.5	3.4	55.3, 66.8
		2	R1	1.0	0.5	0.2	69.2
		3	R1	0.3	0.5	0.8	82.4
	*Left*	1	L1	1.0	1.5	0.1	42.8
9	*Right*	1	R1, R2	0.1	1.7	0.5	59.3, 123.8
		2	R1, R2	0.9	0.0	0.4	80.9, 91.5
10	*Right*	1	R1	0.6	4.2	3.8	57.5
11	*Right*	1	R1	0.1	0.5	4.4	96.6
	*Left*	1	L1, L2	1.8	0.3	1.0	19.5, 45.0
12	*Right*	1	R1	0.7	0.0	0.7	20.5
13	*Right*	1	R2, R3	2.9	0.5	2.6	75.8, 63.4
14	*Right*	1	R3	0.9	0.9	1.2	24.0
		2	R1, R2	0.7	3.6	1.3	86.9, 52.4
	*Left*	1	L2	0.2	0.5	0.7	19.6
		2	L2	1.6	0.2	0.7	65.2
15	*Right*	1	R2, R3	0.1	0.2	1.1	31.5, 39.9
16	*Right*	1	R1, R2	0.4	0.7	0.7	120.1, 66.9
		2	R1, R2	1.2	0.3	0.8	132.2, 85.4
		3	R3	0.4	1.3	1.4	44.2
		4	R1, R2	1.3	0.1	0.8	146.0, 49.5
	*Left*	1	L1	1.7	2.3	1.4	43.3
17	*Right*	1	R2	0.2	4.4	1.2	92.0
		2	R1	0.5	1.8	0.8	81.2
Mean				1.0	1.1	1.8	59.3
Standard deviation				0.8	1.2	1.7	24.6

The fiducial markers were numbered in the superior-inferior direction; for instance, R1 represents the fiducial marker on the right side, which is the most superiorly located.

GTV, gross tumor volume; FM, fiducial marker; R, right; L, left.

**Figure 3:**
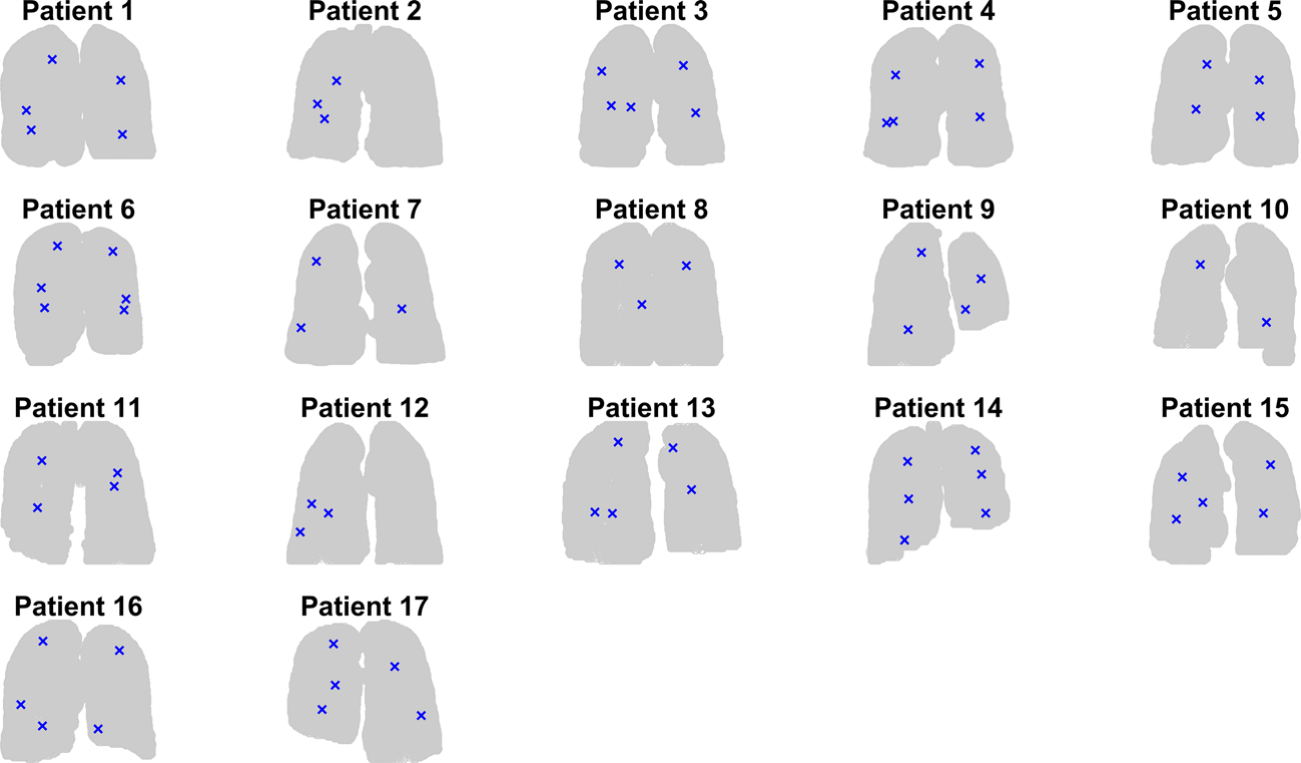
Graphical representation of the location of the intraoperatively implanted fiducial markers on coronal view for each of the 17 patients.

**Figure 4:**
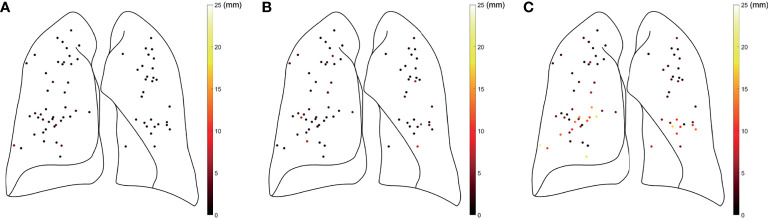
Approximate locations and motion amplitudes (color-coded) in the **(A)** R-L, **(B)** A-P, and **(C)** I-S directions for all the intraoperatively implanted fiducial markers for 17 patients. R-L, right-left; A-P, anterior-posterior; I-S, inferior-superior.

The locations of the IIFMs for each patient are shown in a coronal view in **Figure 3**. In **Figure 4**, the locations of the IIFMs for all patients are approximately plotted with the lungs of representative dimensions and are color-coded depending on the FM motion amplitudes.

### Accuracy of Fiducial Tracking Algorithm: Phantom

**Figure 5:**
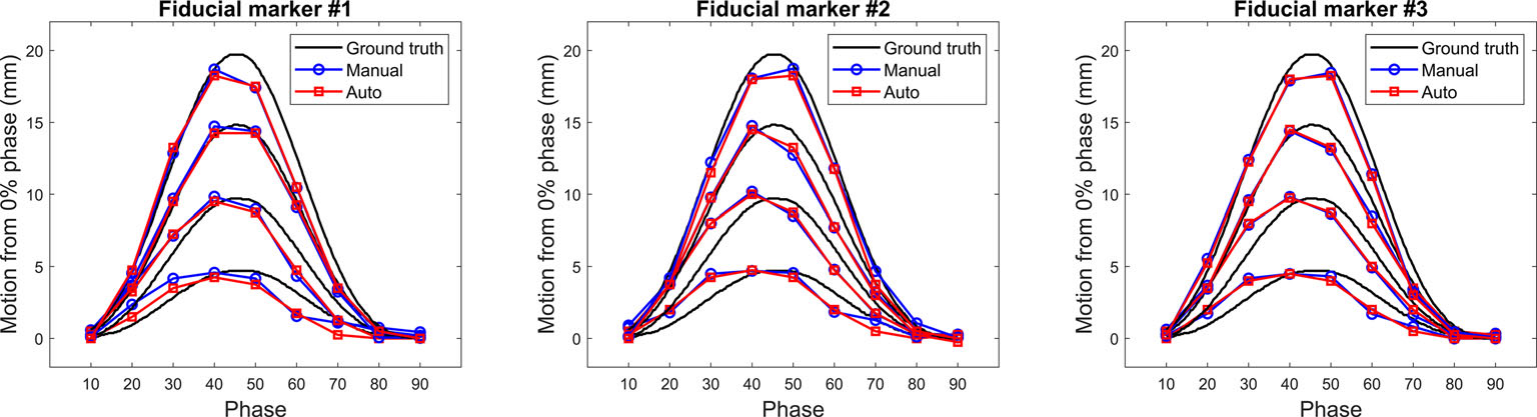
Comparison of the automatically and manually measured motions (0% to the other respiratory phases) of the three fiducial markers inserted into the Quasar phantom with the ground truth motions.

**Figure 5** presents the comparison results of the FM motions calculated using manual and automatic localization methods with the ground truth motions. The manually segmented and registered motions agreed reasonably well with each other, resulting in a difference of 0.2 ± 0.2 mm. The automatically measured motions were smaller than the ground truth motions by, on average, 0.5, 0.3, 0.5, and 1.8 mm for the 5-, 10-, 15-, and 20-mm reference motions, respectively.

### Accuracy of Fiducial Tracking Algorithm: Patient

**Figure 6:**
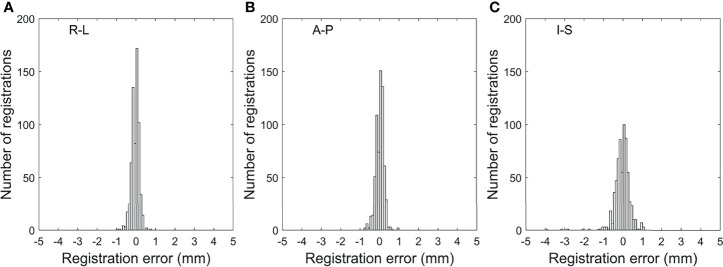
Histograms of the all-phase fiducial marker registration errors by the template matching-based image registration performed for the patient data in the **(A)** R-L, **(B)** A-P, and **(C)** I-S directions. R-L, right-left; A-P, anterior-posterior; I-S, inferior-superior.

The registration errors, which were calculated as the difference between the registered and manually segmented FM positions, are plotted as histograms in **Figure 6**. The absolute registration errors were 0.1 ± 0.1, 0.1 ± 0.1, and 0.3 ± 0.3 mm in the R-L, A-P, and I-S directions, respectively, in all patients. In 12 (1.8%) of the 657 alignments, the registration error was larger than 2 mm in the I-S direction. These relatively large misalignments occurred for the image registrations from 0% to intermediate phases where FMs quickly moved and are thereby abnormally displayed.

The differences between the motion amplitudes that were manually measured and calculated using the template matching algorithm across all the FMs of patients were 0.1 ± 0.1 (range: −0.6 to 0.6), 0.1 ± 0.1 (−0.5 to 0.5), and 0.2 ± 0.2 (−0.9 to 0.9) mm in the R-L, A-P, and I-S directions, respectively.

### Log-Based In-Treatment Motion Analysis

**Figure 7:**
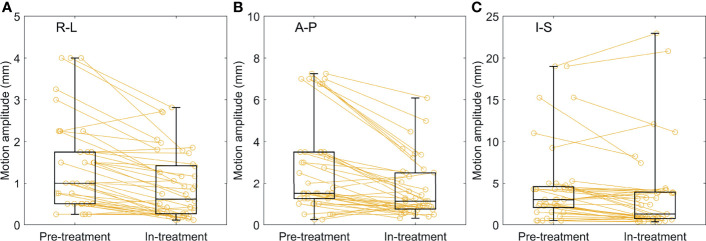
Comparisons of the pretreatment and in-treatment motion amplitudes across 41 treatment fractions of 31 SABR plans in the **(A)** R-L, **(B)** A-P, and **(C)** I-S directions, respectively. The mean values were presented for in-treatment motion amplitudes. SABR, stereotactic ablative radiotherapy; R-L, right-left; A-P, anterior-posterior; I-S, inferior-superior.

**Figure 7** presents paired box plots comparing the pretreatment and mean in-treatment motion amplitudes, which were analyzed using 4D CT and the treatment log file, respectively. The mean in-treatment motion amplitudes were smaller than the pretreatment motion amplitudes, resulting in statistically significant differences of −0.5 ± 0.5, −1.1 ± 1.3, and −1.0 ± 2.0 mm in the R-L, A-P, and I-S directions, respectively (*p* < 0.005 in all directions).

## Discussion

This study demonstrated the feasibility of using the developed ITV_tracking_ margin determination framework to aid in the decision-making process of treating multiple lung metastases using IIFMs. An optimal set of IIFMs was successfully determined for each of the GTV of the patient cohort. An ITV_tracking_ margin of less than 5 mm was required for 41 of 42 patients to cover the motion discrepancy between the GTV and selected FMs. Patient 1, in whom nine lung lesions were defined, is the best example that shows the effectiveness of the IIFM-based RT technique. Without the insertion of FMs during surgery, treating this large number of lung metastases was not practically feasible, requiring implantation of at least 27 FMs for an ideal tracking. However, with the developed framework, nine lung metastases were treated with only five IIFMs by introducing reasonable margins.

To the best of our knowledge, this study is the first to develop an innovative margin determination framework that can facilitate SABR of multiple lung metastases using IIFMs although the clinical relevance of these results is yet to be elucidated and will be reported separately. This novel development suggests a technological combination that supports a challenging treatment, which is performed mainly in an oligo-metastatic setting. First, as suggested by Pop et al. (2), FM placement during surgery can be a promising method to treat potential local recurrence without percutaneous or bronchoscopic insertion. Second, treating multiple lung metastases can be further supported by superior dosimetric characteristics (1) of the CyberKnife plans because of its non-coplanar beam arrangement. Finally, CPAP can reduce the radiation dose to the lungs (10–12). The combination of the aforementioned treatment techniques can facilitate the ablative treatment of multiple lung metastases.

Both phantom and patient studies demonstrated that gold FMs can be localized using the developed fiducial tracking algorithm with a sub-millimeter accuracy despite the limited resolution of 4D CT images (3 mm slice thickness in this study). The motions calculated using the template matching-based fiducial tracking algorithm closely coincided with those manually measured. However, as demonstrated in the phantom study, the motions captured in the 4D CT images (both manually and automatically detected) were similar to but slightly smaller than the ground truth motions. This motion underestimation by 4D CT may be attributed to the motion uncertainty in the phantom. For example, the insert of the phantom containing the FMs was measured to move approximately 19 mm although a motion of 20 mm was simulated. In addition, motion underestimation by 4D CT was reported in a recent study (13). As this investigation focused on the synchronization level between the lung target and IIFMs, underestimation of the respiration motions by 4D CT images may not greatly affect the resulting ITV_tracking_ margin size.

The consistency of the FM motions between simulation and treatment was evaluated using the log file information. The average in-treatment FM motions were significantly smaller than those simulated using the planning 4D CT images. Assuming a consistent synchronization between the target and selected FMs, a smaller motion for GTVs can be theoretically expected and, therefore, a smaller motion discrepancy. In addition, relatively large standard deviations of the intrafraction motion amplitudes indicated that the patient breathed with various amplitudes over the long treatment duration with CyberKnife. Given that the in-treatment motion amplitudes were larger compared with planned motion amplitudes for a small portion of the total treatment time, only a small amount of target miss is expected in the treatment using a selected set of the IIFMs. However, additional margin may be necessary to compensate for further motion discrepancies that possibly occur during treatment.

Careful attention should be paid to planning target volume (PTV) margin determination and patient setup when the CyberKnife Synchrony tracking is used to treat a lung target with fewer FMs than those recommended by the vendor. In particular, with one FM tracked, a small rotation may result in a large displacement of the lung target from the planned position. Therefore, an appropriate selection of PTV margin and/or accurate patient setup are required for the suggested treatment technique. In this study, when the calculated ITV_tracking_ margin was less than 5 mm, a uniform CTV-to-PTV margin of 5–8 mm was applied. Further investigation is warranted for this purpose. Furthermore, a dosimetric comparison between the proposed tracking radiation therapy and VMAT plans needs to be performed with careful consideration on PTV margins for both treatment techniques. A CyberKnife Synchrony plan with an appropriate ITV_tracking_ and PTV margins can be dosimetrically advantageous over a VMAT plan when the patient respiratory motion is relatively large, resulting in a larger target volume for VMAT. When CyberKnife Synchrony and VMAT are available for lung SBRT, a decision-making process should be supported by evaluation of dosimetric benefits. Finally, a long-term report on treatment outcomes and toxicity will be required to provide evidence for the proposed treatment approach.

## Conclusions

The feasibility of using the developed margin determination framework for SABR of multiple lung metastases using IIFMs was demonstrated. With careful consideration of accurate patient setup and appropriate selection of PTV margin, the developed margin determination framework can be successfully used to deliver local ablative dose to multiple lung metastases.

## Data Availability Statement

The raw data supporting the conclusions of this article will be made available by the authors, without undue reservation.

## Ethics Statement

The studies involving human participants were reviewed and approved by Institutional Review Board of Severance Hospital (No. 4-2021-0440, May 26, 2021). Written informed consent for participation was not required for this study in accordance with the national legislation and the institutional requirements.

## Author Contributions

Conceptualization: WK, SP, JC, and C-SH. Methodology: JK, JSK, and C-SH. Software: JK and MH. Validation: JK, MH, and RP. Investigation: KK, HB, and JC. Writing—original draft preparation: JK, JC, and C-SH. Writing—review and editing: JK, JC, C-SH, KK, HB, and SP. Visualization: JK. Supervision: C-SH and SP. All authors contributed to the article and approved the submitted version.

## Funding

This work was supported by the National Research Foundation of Korea (NRF) grant funded by the Korea government (MSIT) (No. NRF-2020R1C1C1005713), by a faculty research grant of Yonsei University College of Medicine (6-2020-0154), and by the Korea Medical Device Development Fund grant funded by the Korea government (the Ministry of Science and ICT, the Ministry of Trade, Industry and Energy, the Ministry of Health & Welfare, the Ministry of Food and Drug Safety) (No. 202012E0102).

## Conflict of Interest

The authors declare that the research was conducted in the absence of any commercial or financial relationships that could be construed as a potential conflict of interest.

## Publisher’s Note

All claims expressed in this article are solely those of the authors and do not necessarily represent those of their affiliated organizations, or those of the publisher, the editors and the reviewers. Any product that may be evaluated in this article, or claim that may be made by its manufacturer, is not guaranteed or endorsed by the publisher.

## Abbreviations

ITV, internal target volume; IIFM, intraoperatively implanted fiducial marker; SABR, stereotactic ablative radiotherapy; FM, fiducial marker; CT, computed tomography; I-S, inferior-superior; CPAP, continuous positive airway pressure; GTV, gross tumor volume; CK, CyberKnife; R-L, right-left; A-P, anterior-posterior; VMAT, volumetric-modulated arc therapy; PTV, planning target volume.

## References

[1] Chan MKH Kwong DLW Law GML Tam E Tong A Lee V. Dosimetric Valuation of Four-Dimensional Dose Distributions of Cyberknife and Volumetric-Modulated Arc Radiotherapy in Stereotactic Body Lung Radiotherapy. J Appl Clin Med Phys (2013) 14:136–49. doi: 10.1120/jacmp.v14i4.4229 PMC5714543 23835388

[2] Pop D Venissac N Bondiau P-Y Mouroux J. Peroperative Fiducial Placement for Postoperative Stereotactic Cyberknife Radiosurgery. Interact Cardiovasc Thorac Surg (2010) 10:1034–6. doi: 10.1510/icvts.2009.227348 20197353

[3] Tsilimigras DI Ntanasis-Stathopoulos I Paredes AZ Moris D Gavriatopoulou M Cloyd JM. Disappearing Liver Metastases: A Systematic Review of the Current Evidence. Surg Oncol (2019) 29:7–13. doi: 10.1016/j.suronc.2019.02.005 31196496

[4] Guckenberger M Lievens Y Bouma AB Collette L Dekker A De Souza NM. Characterisation and Classification of Oligometastatic Disease: A European Society for Radiotherapy and Oncology and European Organisation for Research and Treatment of Cancer Consensus Recommendation. Lancet Oncol (2020) 21:e18–28. doi: 10.1016/S1470-2045(19)30718-1 31908301

[5] McCormick M Liu X Jomier J Marion C Ibanez L. ITK: Enabling Reproducible Research and Open Science. Front Neuroinform (2014) 8:13. doi: 10.3389/fninf.2014.00013 24600387 PMC3929840

[6] Mattes D Haynor DR Vesselle H Lewellyn TK Eubank W. Nonrigid Multimodality Image Registration. In: Sonka M Hanson KM, editors. Medical Imaging 2001: Image Processing (2001). p. 1609–20. doi: 10.1117/12.431046

[7] Mattes D Haynor DR Vesselle H Lewellen TK Eubank W. PET-CT Image Registration in the Chest Using Free-Form Deformations. IEEE Trans Med Imaging (2003) 22:120–8. doi: 10.1109/TMI.2003.809072 12703765

[8] Thévenaz P Unser M. Optimization of Mutual Information for Multiresolution Image Registration. IEEE Trans Image Process (2000) 9:2083–99. doi: 10.1109/83.887976 18262946

[9] Lujan AE Larsen EW Balter JM Ten Haken RK. A Method for Incorporating Organ Motion Due to Breathing Into 3D Dose Calculations. Med Phys (1999) 26:715–20. doi: 10.1118/1.598577 10360531

[10] Goldstein JD Lawrence YR Appel S Landau E Ben-David MA Rabin T. Continuous Positive Airway Pressure for Motion Management in Stereotactic Body Radiation Therapy to the Lung: A Controlled Pilot Study Earlier Versions of This Work Were Accepted for Presentation at the Third ESTRO Forum, April 24-28, 2015, in Barcelona. Int J Radiat Oncol Biol Phys (2015) 93:391–9. doi: 10.1016/j.ijrobp.2015.06.011 26264628

[11] Appel S Weizman N Davidson T Urban D Lawrence YR Symon Z. Reexpansion of Atelectasis Caused by Use of Continuous Positive Airway Pressure (CPAP) Before Radiation Therapy (RT). Adv Radiat Oncol (2016) 1:136–40. doi: 10.1016/j.adro.2016.03.002 PMC5506747 28740882

[12] Di Perri D Colot A Delor A Ghoul R Janssens G Lacroix V. Effect of Continuous Positive Airway Pressure Administration During Lung Stereotactic Ablative Radiotherapy: A Comparative Planning Study. Strahlentherapie Und Onkol (2018) 194:591–9. doi: 10.1007/s00066-018-1278-2 29450589

[13] Steiner E Shieh CC Caillet V Booth J O’Brien R Briggs A. Both Four-Dimensional Computed Tomography and Four-Dimensional Cone Beam Computed Tomography Under-Predict Lung Target Motion During Radiotherapy. Radiother Oncol (2019) 135:65–73. doi: 10.1016/j.radonc.2019.02.019 31015172

